# Infection control in schools for learners with spina bifida: A scoping review

**DOI:** 10.4102/ajod.v13i0.1394

**Published:** 2024-08-09

**Authors:** Sasavona R. Mashamba, Saajida Mahomed, Jacqueline M. van Wyk

**Affiliations:** 1 Discipline of Public Health Medicine, School of Nursing and Public Health, University of Kwazulu-Natal, Durban, South Africa; 2 School of Laboratory Medicine and Medical Sciences, College of Health Sciences, University of KwaZulu-Natal, Durban, South Africa; 3 Discipline of Clinical and Professional Practice, School of Clinical Medicine, University of KwaZulu-Natal, Durban, South Africa; 4 Department of Health Sciences Education, Faculty of Health Sciences, University of Cape Town, Cape Town, South Africa

**Keywords:** spina bifida, learners, schools, infection control programme, guidelines

## Abstract

**Background:**

Despite the widespread prevalence of spina bifida and its impact on individual learners, there is limited information on how infection control is managed in the school environment.

**Objective:**

This scoping review was conducted to map the evidence on infection control programmes at schools for learners with spina bifida (LSB) globally.

**Methods:**

This review followed the Joanna Briggs Institute methodology for the scoping review. A database search was conducted on an internet browser search on MEDLINE, PUBMED, EBSCOhost, Google Scholar, ERIC and Google search engines.

**Results:**

A total of five articles met the inclusion criteria. Most of the schools in the study did not have special facilities or running water to accommodate basic infection control procedures.

**Conclusion:**

Collaboration between policymakers, specialists of spina bifida and infection control in the health sector, the Association of Spina Bifida, specialists in spina bifida under the Department of Education and researchers is needed to improve the lives of LSB through infection control.

**Contribution:**

The study will contribute to the improvement of training of staff working with LSB and the need for more research.

## Introduction

Spina bifida (SB) is a neural tube defect and congenital abnormality in which the spinal cord and vertebrae do not form completely, resulting in abnormality during the development of the neural tube (Bannink, Idro & Van Hove 2016b; Sandler 2004). The neural tube usually closes by the fourth week of pregnancy. In SB, the neural tube does not close completely and leaves the baby’s delicate spine open to possible injury (Wisconsin 2019). This results in permanent damage to the spinal cord and the nervous system and can result in paralysis of the lower limbs or problems with bowel and bladder function (World Health Organization [WHO] 2012). The three most common presentations of SB include myelomeningocele, meningocele and spina bifida occult (Centers for Disease 2020). About 80%–90% of babies with SB also develop hydrocephalus, a condition that causes a fluid build-up inside the head causing an increase in pressure which leads to the skull expanding to larger-than-normal size (WHO 2012).

Learners with spina bifida (LSB) face many challenges in resource-scarce countries; most lack access to surgery or help to manage their ongoing disability and symptoms (Shaer, Chescheir & Schulkin 2007). The majority have some degree of paralysis, which affects their mobility, bowel and bladder control (Northrup & Volcik 2000). Almost all individuals with SB have some degree of bladder and bowel dysfunction because the low sacral nerves that innervate the distal bowel, anal sphincter, bladder, and internal and external bladder sphincters are affected (Shaer 1997). Approximately 70% of all LSB will require some form of drug therapy to control an overactive bladder and they will have to practice clean intermittent catheterisation on a lifelong basis (Nordqvist 2014).

Infections associated with SB often interfere with regular school attendance of LSB (Potter Patty & Zabel 2009). These infections include infection of the urinary tract that results from bladder paralysis, and wound infection because of a lack of protective pain sensation (Swaroop & Dias 2009). The frequency of such infections means that almost all LSB require lifelong antibiotic treatment to prevent infection (Nordqvist 2014). Successful integration of a child with SB into the school environment requires attention to various aspects including the physical structure, architecture of the school building, the availability of support and the curriculum (Spina Bifida Association 2015). Learners with myelomeningocele need to learn mobility skills and often require the aid of crutches, braces and wheelchairs (Spina Bifida Association 2015). Learners may require assistance from specialised caregivers including classroom assistants, resource teachers and school nurses to support them in the school environment (Governey, Culligan & Leonard 2014). It is, thus, necessary that LSB are managed in a comprehensive manner and with help and support from a multidisciplinary team (Uehara 2002, Spina Bifida Association 2018).

Schools for LSB are advised to implement infection control programmes to manage infections among learners. Implementing such programmes can also improve the quality of life for learners by reducing infection. The success of such a programme varies depending on the context, policy, type of schools, beliefs and attitudes of staff, and how they implement the programme in their setting.

Despite the widespread prevalence of SB and its impact on individual learners, there is limited information on infection control in the school environment to manage the disease. This scoping review was conducted to map the evidence on infection control programmes at schools for LSB globally.

### Materials and methods

Given the lack of information on this topic, a scoping review was regarded as the most appropriate strategy to map global evidence on infection control for LSB in a school setting. A scoping review protocol was developed before the review, but it was not registered. This review was guided by a reporting guideline for systematic reviews (PRISMA Extension for Scoping Reviews) (Tricco et al. 2018). The review followed the Joanna Briggs Institute methodological approach for scoping reviews (Peters et al. 2015) and includes eight key steps:

- Identifying the research question.
- Identifying relevant studies.
- Study selection.
- Charting the data.
- Collating, summarising and reporting the results.
- Analysis of the evidence.
- Presentation of results.
- Summarising the evidence, reviewing and making a conclusion on the findings.

### Identifying the overall research question

This review was conducted to map the global evidence on infection control programmes at schools for LSB. The following research objectives were formulated to guide the review:

- What types of schools accommodate LSB?
- What obstacles do LSB face in accessing infection control measures within school settings?
- What programmes or guidelines inform how infection control is practised at schools for LSB?
- What is reported about the knowledge, attitudes and practices of staff working at schools that accommodate LSB?

### Identifying relevant studies

The search strategy was informed and guided by the PCC (Population, Concept and Context) framework (Peters et al. 2017).

P – Population: LSB from 5 to 25 years of age.

C – Concept: Infection control programmes in schools.

C – Context: International or globally.

Eligibility criteria were established before the study and guided the selection and screening process (Table 1).

**TABLE 1:** Inclusion and exclusion criteria.

Inclusion criteria	Exclusion criteria
Articles published between 2000 and 2020, in any language and reporting on: Schools accommodating LSB who are 5 to 25 years of age. Guidelines and recommendations to control infection in schools for LSB. Availability of infection control programmes in schools for LSB. Guidelines regarding the availability of human resources, and infrastructure to manage LSB. Training for teachers, care workers and professionals caring or teaching LSB.	Articles with any of the following characteristics: Infection control programmes for SB not based in educational settings. General infection control programmes, not specific to LSB.

LSB, learners with spina bifida.

#### Search strategy

**FIGURE 1:**
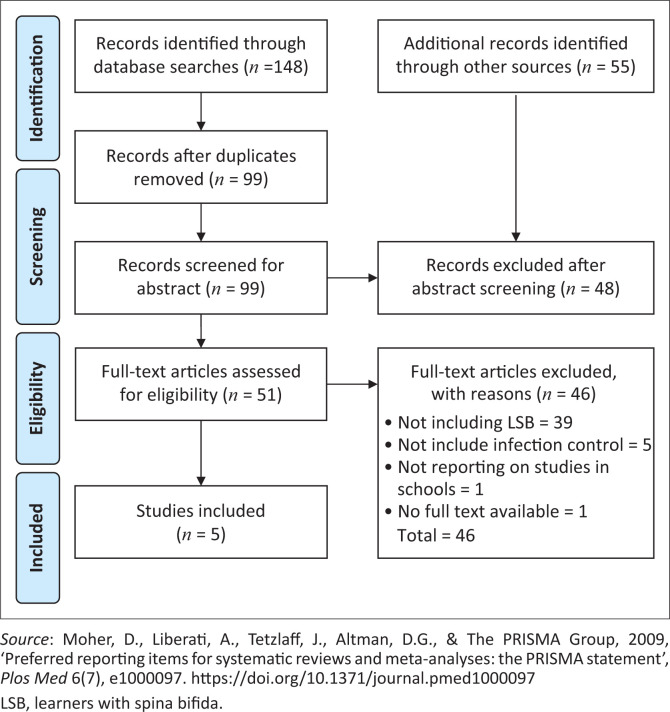
PRISMA flow chart showing literature search and selection of studies.

Searches conducted on MEDLINE, PUBMED, EBSCO host, Google Scholar and ERIC identified 148 articles on the topic. A search conducted by Google search engine found 55 articles. Google Scholar and Google search engine were searched separately. Google Translate was used to translate non-English articles. Duplicates were removed and 99 articles remained which were included in abstract screening. After screening the abstracts, five articles were found to be eligible for inclusion in full article screening (Figure 1).

Searching the literature for eligible articles consisted of two stages. The first search was conducted in May 2020 for studies published from 2000 to 2020. The following search terms were used: infection control, spina bifida, available schools, infection programmes and guidelines. The searches were conducted in Google Scholar, EBSCO host, PUBMED, ERIC and MEDLINE.

A second search was conducted because the first search did not yield sufficient results. The search was conducted in October 2020. The search strategy was piloted on PUBMED, Google Scholar, EBSCO host, PUBMED, ERIC and MEDLINE using the following search terms: (‘spinal dysraphism’[MeSH Terms (Medical Subject Headings terms were used and included to identify studies relevant to the topic) OR (‘spinal’[All Fields] AND ‘dysraphism’[All Fields]) OR ‘spinal dysraphism’[All Fields] OR (‘spina’[All Fields] AND ‘bifida’[All Fields]) OR ‘spina bifida’[All Fields]) AND (‘child’[MeSH Terms] OR ‘child’[All Fields] OR ‘learners’[All Fields]) AND (‘schools’[MeSH Terms] OR ‘schools’[All Fields] OR ‘school’[All Fields]) AND (‘infections’[MeSH Terms] OR ‘infections’[All Fields] OR ‘infection’[All Fields]) AND (‘communicable diseases’[MeSH Terms] OR (‘communicable’[All Fields] AND ‘diseases’[All Fields]) OR ‘communicable diseases’[All Fields] OR (‘infectious’[All Fields] AND ‘disease’[All Fields]) OR ‘infectious disease’[All Fields]) AND (‘prevention and control’[Subheading] OR (‘prevention’[All Fields] AND ‘control’[All Fields]) OR ‘prevention and control’[All Fields] OR ‘control’[All Fields] OR programme[All Fields] AND (‘guideline’[All Fields] OR ‘guidelines as topic’[MeSH Terms] OR ‘guideline’[All Fields]) OR ‘measures’[All Fields]) OR ‘measure’[All Fields]).

### Study selection

Titles were screened using the following key terms: learners or school-going LSB or spinal dysraphism, special needs, physical disabilities, infection control, management, prevention programme, measures, staffing, personnel, support staff, infrastructure, schools, centres for learning, facilities, placements, guidelines, recommendations, schools for LSB, management of SB and spinal dysraphism at school, guidelines for the management of SB and infection control for LSB and dysraphism and school personnel for LSB.

The results from the database searches were exported into a single library in an EndNote X9 reference manager programme. The selection of articles incorporated two stages: the first stage involved screening all titles and abstracts generated on the searches by two authors independently. All eligible articles were retained by applying the inclusion and exclusion criteria as detailed earlier in the text. In the second stage, the full texts of all eligible articles were reviewed by the same two authors to determine whether the article should be included in the study. Any disagreements between the first two reviewers were discussed and where necessary, resolved by the third reviewer.

### Charting data

If an article was eligible for inclusion, details about the study itself were charted; including author and date, the title of the study, publication, aims of the study, study setting, study population, sampling method, study design, data collection methods, data analysis and the most relevant findings in response to the research questions. The extracted data were then entered into a Microsoft Excel spreadsheet.

### Collating, summarising and reporting the results

The findings were gathered and summarised, and an explanation of data extracted from the included studies was analysed using content analysis.

### Analysis of the evidence

The findings were analysed to answer the research questions and address the purpose of the review. The synthesis of results consisted of quantitative analysis (e.g. frequency analysis) of the scoping review, conduct (i.e. methodological steps), and qualitative analysis (i.e. content analysis) of the components of the research purpose. For the qualitative analysis, an Excel spreadsheet was developed in which relevant findings were categorised. A narrative synthesis was performed to map data on infection control for LSB.

### Ethical considerations

An application for full ethical approval was made to the the University of KwaZulu-Natal Biomedical Research Ethics Committee and ethics consent was received on 31 October 2019. The ethics approval number is BREC/00000854/2019.

## Presentation of results

The included studies provided feedback on each of the research objectives: types of schools accommodating LSB, obstacles faced by LSB in accessing infection control measures within the school settings, report on infection control policy, guidelines and procedures for schools accommodating LSB, report about knowledge, attitudes and practices of staff working at schools accommodating LSB.

### Characteristics of included studies

Three of the five studies were conducted in low-middle-income countries, two from Uganda (Bannink, Idro & Van Hove 2016a; Bannink et al. 2016b) and one from India (Mathew 2006). The remaining two studies were conducted in the United States of America (USA) (Katrancha 2008) and Ireland (Governey et al. 2014). The studies used qualitative (Bannink et al. 2016b, Katrancha 2008), descriptive quantitative (Mathew 2006) and mixed methods (Bannink et al. 2016a; Governey et al. 2014) research designs (Table 2).

**TABLE 2:** Characteristics of included studies.

Author and date	Title	Country of study	Research design	Research Methodology	Sample and sample size	Aims of the study	Themes mentioned	Sub-themes mentioned
Bannink et al. (2016b)	Teachers’ and parents’ perspectives on inclusive education for children with SB in Uganda	Uganda	Qualitative	Qualitative semi-structured Interviews, observation	LSB (*n* = 97) Age 4–14 years (*n* = 63) Parents (*n* = 30) Teachers (*n* = 30)	Describe the primary school setting for LSB in the central region of Uganda and explore accessibility and inclusion with parents and teachers.	Procedures	Infrastructure
Bannink et al. (2016a)	I like to play with my friends: children with SB and Belonging in Uganda	Uganda	Mixed method	Qualitative semi-structured interviews, observation, quantitative functioning scales measurement	LSB (*n* = 97) Age 4–14 years (*n* = 63) Siblings (*n* = 39) Parents (*n* = 139)	Describe experiences of belonging for LSB and their families living in Uganda	Procedures	-
Governey et al. (2014)	The health and therapy need of LSB in Ireland. Dublin: Temple Street Learners’ University Hospital	Ireland	Mixed method	Quantitative questionnaires, qualitative interviews, focus group	LSB (*n* = 14) Age 8–18 years (*n* = 14) Service providers (*n* = 247) Parents or guardians (*n* = 155)	Determine the health, therapy, and service needs of LSB from the perspective of parents and service providers	Policy and procedures	Infrastructure
Katrancha (2008)	Clean intermittent catheterisation in the school setting	USA	Qualitative	Case study	LSB (*n* = 1) Age 10 years (*n* = 1)	Discuss the role of the school nurse	Guidelines, procedures	Human resource
Mathew (2006)	Study to assess the compliance and selected factors affecting compliance to clean intermittent catheterisation in LSB to develop guidelines for home management in the Indian setting	India	Quantitative	Baseline proforma, semi-structured, structured interview, attitudes scale and observation	LSB (*n* = 30) Children (*n* = 30) Age 1–18 years (*n* = 30) Parents (*n* = 30)	Assess the compliance and selected factors affecting compliance to clean intermittent catheterisation LSB	Procedures	Infrastructure

LSB, learners with spina bifida.

## Collating, summarising and reporting the results

### Types of schools accommodating learners with spina bifida

All the included studies reported on schools for LSB (see Table 3). Learners were attending private or mainstream pre-primary, primary or secondary schools; some were enrolled in special schools (Bannink et al. 2016a, 2016b; Governey et al. 2014; Katrancha 2008; Mathew 2006). In the study from India, 11 (36%) LSB were accommodated in mainstream primary schools, three (10%) in middle school, one (3.3%) in high school and one (3.3%) in a special school (Mathew 2006). While in the USA, LSB attended mainstream pre-primary schools (Katrancha 2008), 89% of LSB in Ireland attended mainstream schools (18 learners in preschool, 47 in primary school and 14 in secondary school), nine (10%) learners attended special education and one learner (1%) was home-schooled (Governey et al. 2014). Some reports included learners who attended public schools that offered special education (Governey et al. 2014). Five were at the pre-primary school level, and four each were enrolled at primary school and high school levels. The mean age of the learners was 7 years with a range from 2.4 years to 16.3 years (Governey et al. 2014). In contrast, in the study conducted in Uganda, 26 (63%) LSB were attending private nursery schools, 11 (26.8%) were in private primary schools and 4 (9.7%) were in private secondary schools (Bannink et al. 2016b). In another study conducted in Uganda, 50 (36.8%) were in private nursery schools, 22 (16.2%) were in private primary schools and four (2.9%) were in private secondary schools (Bannink et al. 2016a). Their ages ranged from 4 to 14 years (Bannink et al. 2016a). The number of pupils per class in private schools varied between 28 and 69 learners (Bannink et al. 2016b). The number of learners per class was not reported in the study from Ireland (Governey et al. 2014).

**TABLE 3:** Type of schools for learners with spina bifida.

Country	Type of school	Number of LSB enrolled
India	Mainstream primary school	11
	Mainstream Middle School	3
	Mainstream High School	1
	Special School	1
Ireland	Mainstream Preschools	18
	Mainstream Primary Schools	47
	Mainstream Secondary Schools	14
	Special Education Pre-Primary Schools	5
	Special Education Primary	2
	Special Education High Schools	2
	Home School	1
Uganda	Private Nursery Schools	50
	Private Primary School	22
	Private Secondary School	4
Uganda	Nursery School	26
	Primary School	11
	Secondary School	4

Note: Mainstream Pre-Primary School from USA did not report number of LSB enrolled.

LSB, learners with spina bifida.

### Obstacles faced by learners with spina bifida in accessing infection control measures within school settings

The studies from Uganda, India and Ireland reveal significant obstacles faced by LSB in accessing infection control programmes in school settings as well as accessing education (Bannink et al. 2016a, 2016b; Governey et al. 2014; Mathew 2006). In Uganda, a mere 65% of the LSB study population attended school (Bannink et al. 2016a), with many dropping out because of peer bullying, lack of income and societal stigma (Bannink et al. 2016a). Wheelchair inaccessibility, absence of continence management programmes and inadequate facilities resulted in the exclusion of LSB from schools (Bannink et al. 2016b).

In India, 60% of LSB attended school, but challenges persisted. Inadequate water and toilet facilities affected catheterisation practices, highlighting infrastructure deficiencies (Mathew 2006). Ireland demonstrated a higher attendance rate (89%), emphasising regional variations (Governey et al. 2014). However, across Uganda, India and Ireland, physical and logistical challenges in accessing school facilities were consistent (Bannink et al. 2016b; Governey et al. 2014; Mathew 2006. In Ireland, access to mainstream schooling for LSB depended on support for their physical and toilet needs (Governey et al. 2014).

Physical access to schools was generally poor, particularly in Uganda, where classrooms, playgrounds, toilets and changing areas posed difficulties (Bannink et al. 2016b). Wheelchair access, especially during the rainy season, and the lack of functional ramps further restricted mobility (Bannink et al. 2016b). Additionally, LSB lacked access to essential facilities such as libraries and sports fields. Boarding facilities in Uganda also lacked wheelchair access, with no bathroom access for LSB (Bannink et al. 2016b).

Furthermore, the unhygienic nature of communal school toilets in Uganda made practising intermittent catheterisation challenging (Bannink et al. 2016b). Only a few schools provided library facilities; the study highlighted the overall inadequate space and hygiene for practising essential medical procedures (Bannink et al. 2016b). While some schools allowed LSB access to staff toilets (Bannink et al. 2016b), the broader infrastructure challenges underscored the pervasive difficulties faced by LSB in realising their right to education and healthcare. These findings emphasise the urgent need for inclusive policies, improved infrastructure and societal awareness to overcome the substantial barriers impeding the education and well-being of LSB.

### Infection control policy, guidelines and procedures within the schools

Two of the studies reported on the presence of policies for physical access and inclusion of LSB, but the policies were not implemented (Bannink et al. 2016b; Governey et al. 2014). In Ireland, Governey et al. reported that the United Nations Convention on the Rights of People with Disabilities set by the United Nations (Training Guide 2014) stated that all countries should provide full access to school facilities to learners with disabilities and ensure that they have full enjoyment of their rights (Governey et al. 2014). Parents, however, raised concerns about the services provided to their children such as poor physical access and exclusion from the school environment (Governey et al. 2014).

In Uganda, teachers reported that their schools followed the regulations and curriculum of the Inclusive Education Policies, set by the Government of Uganda (Uganda 2015), which regulated schools for learners with disabilities (Bannink et al. 2016b). Despite these regulations, teachers reported difficulties for LSB to participate in classroom activities because of a lack of access to buildings, large student numbers, limited resources and teaching material (Bannink et al. 2016b).

In the USA, Katrancha reported on guidelines for clean intermittent catheterisation in the school setting (Katrancha 2008). The guidelines recommend that clean intermittent catheterisation be performed four times daily, twice during the school day and in a private place (Katrancha 2008). The first procedure should be undertaken mid-morning and the second mid-afternoon (Katrancha 2008). Class teachers need to coordinate specific times to minimise the loss of instructional time (Katrancha 2008). The guidelines stipulate that the equipment to practice clean intermittent catheterisation includes a sink for handwashing, catheters, lubricant, alcohol-free wipes or washcloths, a handheld mirror, a container, or toilet to catch urine, mild liquid soap to wash the catheter, a syringe for flushing the catheter and container to store the catheter (Katrancha 2008).

### Knowledge of staff working at the schools for learners with spina bifida

Two of the five studies reported that teachers did not offer diversified teaching (Bannink et al. 2016a, 2016b). One of the five studies reported on meetings with parents to discuss individual education plans and the financial burden of using incontinence products (Katrancha 2008). A study conducted in the USA reported on the mother of an LSB who had a meeting with a school nurse, guidance counsellor, classroom teacher and special education supervisor to update her on the individual education plan and obtain more information about the learners’ health status (Katrancha 2008). The school nurse also discussed the development of and the need for independence with the mother (Katrancha 2008). In addition, the school nurse assisted the mother in devising a plan for clean intermittent catheter programmes (Katrancha 2008).

### Attitudes of staff employed at the schools for learners with spina bifida

Some teachers in the Ugandan study were unwilling to assist LSB (Bannink et al. 2016b). The study reported that LSB were bullied by other learners and that teachers did little to address the bullying (Bannink et al. 2016b). Teachers in private schools felt that parents should remove LSB from school (Bannink et al. 2016b). Only half of the teachers believed that it was part of their responsibility to prevent an LSB from being bullied in class, while others believed it was the responsibility of the school management and parents to deal with bullying behaviour (Bannink et al. 2016b). Some teachers blamed the parents for enrolling a learner who was unable to walk and who had no wheelchair (Bannink et al. 2016b). Learners with spina bifida were then teased when they had to crawl to get around (Bannink et al. 2016b). The teachers mostly thought it was the learners themselves or their parents’ responsibility to perform clean intermittent catheterisation even in the school environment (Bannink et al. 2016b). In some schools, the teacher or a nurse did, however, assist the learners with clean intermittent catheterisation (Bannink et al. 2016b).

### Practices of staff working at the schools for learners with spina bifida

Two of the five studies reported on incontinence in Ireland and Uganda (Bannink et al. 2016b; Governey et al. 2014). Catheterisation and toileting were often provided by a special needs assistant or, occasionally, by a parent (Governey et al. 2014). In Uganda, while parents usually assisted learners with toileting, some teachers or nurses were also reported to have assisted (Bannink et al. 2016b). Teachers reported that the mothers of some of the learners who were incontinent came to the school to help with their toilet needs (Bannink et al. 2016b). However, the mothers sometimes arrived too late which caused accidents and urine leakage causing other learners to complain (Bannink et al. 2016b). In one of the studies where the teachers lacked teaching assistants, other learners were asked to help LSB in the classroom setting (Bannink et al. 2016b). The tasks performed by the peers included helping them leave the classroom or copying notes where their writing was difficult, or slow or where they had missed classes for medical reasons (Bannink et al. 2016b). Learners with spina bifida were never asked to help other learners in class (Bannink et al. 2016b).

## Summarising the evidence and concluding remarks

A significant strength of this review was the comprehensive and systematic searches of five databases for published literature on school facilities for LSB, recommendations on infection control in the schools, availability of infection control programmes in the school, availability of human resources and infrastructure to manage LSB, and training for teachers, care workers and professionals caring for or teaching LSB. We believe that all relevant information has been included. Most of the studies used descriptive and or mixed-method study designs. Most studies were conducted in low-middle-income countries, and relatively few studies were conducted in the USA.

### Theme 1

Although the studies reported on the studies for physical access and inclusion of LSB, none of the publications discussed widespread adherence to such policies or guidelines. For example, none reported on the ratio of students to teachers that should be applied in schools for LSB. Most reports included initiatives by parents who arranged for intermittent catheterisation themselves (Bannink et al. 2016b; Governey et al. 2014). The findings from a study at primary and secondary mainstream schools in the United Kingdom suggest that staff are uncertain about their roles and responsibilities and lack appropriate training and support to perform healthcare roles (Mukherjee, Lightfoot & Sloper 2000). It is important to ensure that all stakeholders involved in the education and care of LSB are well-trained and have the relevant resources to achieve academic success (Wairimu 2018). Most schools did not have special facilities, or running water to accommodate basic infection control procedures (Bannink et al. 2016a, 2016b; Governey et al. 2014; Mathew 2006). Only one school allowed learners to use the teachers’ bathroom (Bannink et al. 2016b).

One study in the USA mentioned the existence of guidelines for clean intermittent catheterisation performed with the assistance of a nurse (Katrancha 2008). The other three studies that mentioned infection control in Ireland and Uganda did not mention any guidelines that were followed to practice infection control (Bannink et al. 2016a, 2016b; Governey et al. 2014). None of the studies mentioned policies on infection control for LSB in the school setting.

Another study noted that guidelines for healthcare procedures in schools are intended to enhance the educational experiences of learners with healthcare needs by guiding school nurses, teachers and other staff (Southhall 2017). Policymakers should collaborate with specialists of SB in the health sector, researchers and the Association for Spina Bifida to develop guidelines recommendations, and policies for infection control for LSB in the school setting and ensure that they are implemented and monitored.

### Theme 2

Only one study mentioned the presence of a nurse (Katrancha 2008) and none of the studies mentioned additional training or support for teachers to address coping with an LSB in their classroom. The study conducted in the USA has shown that school-based nursing support helps to build self-esteem and allows LSB greater independence at school (Katrancha 2008). Much of the assistance and many of the services that LSB need fall under social services, so there is a need to collaborate and coordinate with other professionals in this sector (Gaintza, Ozerinjauregi & Aróstegui 2018). The fact that LSB are perceived to have a learning problem is a major concern and an important issue to address to ensure appropriate support in the classroom (Kalucy, Bower & Stanley 1996). The lack of teachers’ skills in adapting the curriculum to meet a range of learning needs was identified as a contributory factor to hampering the progress of inclusive education in another South African study (Chataika et al. 2012).

The lack of education about the condition, especially in resource-constrained countries, has led to negative attitudes of teachers towards the learners, their delayed reactions to prevent bullying and poor understanding of how to help LSB reach their full potential (Bannink et al. 2016b). Other studies noted that negative attitudes may result from teachers’ inexperience in teaching learners with special needs (McLeskey et al. 2001; Vaughn et al. 1996).

Parents and learners mentioned that learners with disabilities are safer in special schools specifically for learners with disabilities because of the intolerant attitudes of other learners and school staff (Hinton & Kirk 2015). Teachers must be educated about LSB, their education, their disability, and their need to gain confidence in their work and develop a positive attitude. Teacher attitude towards LSB is an important factor in the quality of education received by LSB (Adulaziz Alsolami 2023).

This review has provided evidence of the limited knowledge and lack of training about spina bifida and infection control for teachers in low-middle-income countries. In contrast, research indicates that LSB experiences a general lack of accessibility to facilities and high rates of bullying (Bannink et al. 2016a, 2016b; Governey et al. 2014; Mathew 2006). This highlights the lack of enforcement of policies. Although there is research on spina bifida, there is little research on infection in LSB in a school setting.

It is still unclear whether the responsibility to provide resources needed for infection control rests with the parents or the school. Further research is necessary to investigate the responsibility of providing resources for infection control on LSB.

## Limitations

There were some differences between reviewers at full article screening; however, they were resolved by a third reviewer and an agreement was reached. The assessment of bias was not conducted on the studies included. The effectiveness of the search strategy used to retrieve relevant studies is crucial. Some relevant studies may have been missed because of the choice of databases and keywords. Scoping reviews might be susceptible to publication bias, as they often rely on published studies. The review’s findings may be limited by the period within which the included studies were published. New evidence might have emerged since then, impacting the overall conclusions. A librarian was not involved in the development of the search strategy.

### Conclusion

The findings of the review have provided evidence of poorly functioning educational systems that do not serve the needs of LSB in low-middle-income countries. Teachers lack sufficient knowledge of infection control in LSB and, thus, there are inadequate infection control practices in schools that accommodate LSB. Training of staff at schools with LSB is required. These schools need the appropriate resources, including health professionals, special needs assistants and social services professionals, to cater to the needs of LSB.
